# A new *ecology-on-a-chip* microfluidic platform to study interactions of microbes with a rising oil droplet

**DOI:** 10.1038/s41598-019-50153-9

**Published:** 2019-09-24

**Authors:** Andrew R. White, Maryam Jalali, Jian Sheng

**Affiliations:** Department of Engineering, Texas A&M University-Corpus Christi, Corpus Christi, TX 78412 USA

**Keywords:** Microbial ecology, Mechanical engineering

## Abstract

Advances in microfluidics technology has enabled many discoveries on microbial mechanisms and phenotypes owing to its exquisite controls over biological and chemical environments. However, emulating accurate ecologically relevant flow environments (e.g. microbes around a rising oil droplet) in microfluidics remains challenging. Here, we present a microfluidic platform, i.e. *ecology-on-a-chip* (*eChip*), that simulates environmental conditions around an oil droplet rising through ocean water as commonly occurred during a deep-sea oil spill or a natural seep, and enables detailed observations of microbe-oil interactions at scales relevant to marine ecology (i.e. spatial scales of individual bacterium in a dense suspension and temporal scales from milliseconds to weeks or months). Owing to the unique capabilities, we present unprecedented observations of polymeric microbial aggregates formed on rising oil droplets and their associated hydrodynamic impacts including flow fields and momentum budgets. Using the platform with *Pseudomonas*, *Marinobacter*, and *Alcarnivorax*, we have shown that polymeric aggregates formed by them present significant differences in morphology, growth rates, and hydrodynamic impacts. This platform enables us to investigate unexplored array of microbial interactions with oil drops.

## Introduction

The interactions of active colloids (e.g. motile microbes) with liquid-liquid interfaces (e.g. a rising oil droplet) has received increasing attentions across scientific disciplines (e.g. microscale transport, microbiology, and tissue engineering, *etc*.) and applications (e.g. drug delivery, biofouling prevention, and pharmaceuticals synthesis etc.), especially, in the wake of *Deepwater Horizon* disaster. Due to wellhead injection of dispersant, clouds of insoluble oil (e.g. n-alkanes) droplets (<1 mm) were generated at depth and rose through the water column^1^. Contrary to the consensus, field observations have revealed that these droplets were “trapped” in a deep-sea plume between depths of 900 and 1300 m^2–6^ and coincided spatially and temporally with microbial blooms^2,7–15^ and a sedimentation event^5,16–22^, *a*.*k*.*a*. “dirty blizzard”. These unprecedented phenomena raised debates on alternative fates (i.e. biodegradation and sedimentation) of these droplets instead of surfacing, and particularly the underlying mechanisms. Despite significant efforts, these questions remain unanswered and key processes unresolved, which highlights our lack of knowledge in complex oil-colloid interactions under varying mechanical, chemical and biological oceanic environments subject to a rising oil droplet (e.g. temperature, flow shear, microbes and their secretions).

This knowledge gap can be attributed to the lack of observations and proper experimental techniques that are capable of observing microorganisms interacting with a rising oil droplet (e.g. ~100 *μ*m) covering a wide range of spatiotemporal scales. For instance, to adequately resolve microbial interactions with oil-water interfaces, observations must be conducted microscopically with spatial resolutions sufficiently high to resolve individual microbes (e.g. ~*μm*) and temporal resolutions short enough to image their motion (e.g. ~*ms*); meanwhile in order to bridge these individual-based mechanistic observations to population-scale real world phenomena (e.g. natural assemblage responses to oil spills or natural seeps), microcosm experiments must simulate real world physical, chemical and biological environments at adequate length (e.g. microbe length scales) and time scales (e.g. days or weeks for biodegradation processes)^23^. Microfluidics is a promising platform for such investigations considering its success in other microbiological studies such as: bacteria mobility and motility^24–26^; chemotaxis and thermotaxis^27,28^; biofilm and streamer formation^29^; quorum sensing and community dynamics^30^; anti-biofouling substrates^31^; and biofilm rheology^32^. However, existing techniques fail to emulate physical, chemical and biological environments near a moving interface (e.g. under flow shear), especially to provide long-term microscopic observations around a moving droplet, i.e. the droplet would only remain in the stationary field of view for seconds, which is too short to bear any ecological relevance.

To circumvent the problem, three-dimensional tracking microscopy^33^ that actively follows moving objects has been developed but with limited applications due to its complexities. The alternative approach is to “hold” the droplet/particle stationary and apply a reciprocal flow toward it. Controlling and trapping particles and droplets on-chip has itself received much attention in the past. Previous methods include use of geometry^34^, optical tweezers^35^, electrowetting^36^, and acoustic trapping^37^, however, none of them provide suitable controls for the study of oil droplets. Using channel geometry to hold an oil droplet steady will greatly affect the hydrodynamics around the droplet. Optical tweezers, ideal for manipulating particles up to ~10 *μ*m, are too small to trap droplets. Additionally, the technique can cause cell damage, as can electrowetting techniques. Although acoustic tweezers are capable of trapping a ~100 *μ*m oil droplet, the intense modulation of pressure field around the trap damages passing bacterial cells and significantly alters the hydrodynamic environment around the droplet. Thus, there are currently no suitable methods to trap an oil droplet on-chip while leaving suspended bacteria undisturbed to emulate the microecosystem around a rising oil droplet in the water column.

In this manuscript, we describe an experimental microcosm platform, namely *ecology-on-a-chip* (*eChip*), for studying microbial interactions with a rising oil droplet at ecologically relevant length and time scales. The method involves generating and trapping a micro-scale oil droplet in a microfluidic channel while allowing suspended bacteria to naturally flow past and interact with the droplet in conditions that mimic real oceanic environments. The *eChip* allows us to obtain high spatial (<1 *μm*) and large temporal (>weeks at the resolution of 1 ms) observations and leads to previously unattainable insights of microbial behaviors at the oil-water interface.

## Results and Discussion

### Microfluidic channel design

The microfluidic channel was designed to fulfill the following functions: (i) it must be capable of producing a single sub-millimeter oil droplet (e.g. 100 *μ*m) on-chip; (ii) it must be capable of trapping the generated droplet in a location while preserving its circular shape, maintaining an oleophobic contact angle with the top and bottom channel walls, and thus closely emulating the hydrodynamics around a rising micro-droplet; and (iii) it must be able to withstand the long-term observation that lasts weeks. The disposable *eChip* consists of a polydimethylsiloxane (PDMS) microchannel bonded to a glass microscope slide with air plasma. The channel was fabricated using traditional soft lithography techniques (see Methods), which ensure the easiness of applications and transfer of the technology.

#### Principle of single droplet generation

**Figure 1:**
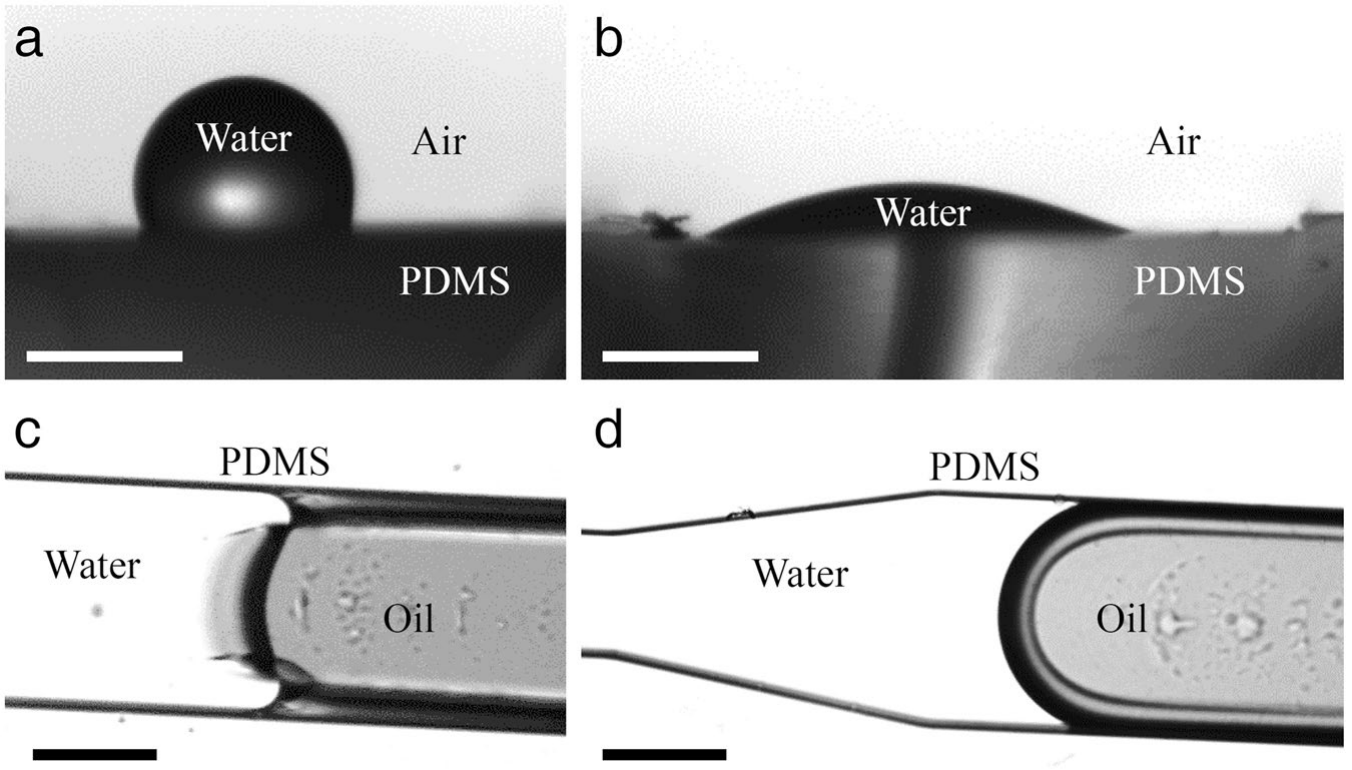
Effects of the hydrophilic polyelectrolyte coating. A DI water sessile drop rests on (**a**) an untreated PDMS surface with a contact angle of 110°; on (**b**) a deconstructed microchannel functionalized using the polyelectrolyte multilayer with a contact angle of 20°. Crude oil water interface in an (**c**) untreated and (**d**) treated microchannel filled with water resulting in oleophilic (**c**) and oleophobic (**d**) contact angle respectively. Scales: 1 mm in (**a**,**b**); 100 μm in (**c**,**d**).

Although generating droplets on-chip has a long history^38^, fewer methods in literatures have successfully generated a single droplet with controlled size^39^. Additionally, generating an oil droplet in a continuous aqueous phase, as opposed to an aqueous droplet in a continuous oily phase, is challenging due to the affinity of an oil droplet to spread over the PDMS hydrophobic microchannel walls (Fig. 1a) and consequently fails to maintain its circular shape and oleophobic contact angle. To overcome this problem, the inner walls of *eChip* can be made hydrophilic to result in the oleophobicity necessary for the dispersed oil. One common practice is to use oxygen plasma to activate PDMS and glass substrates of the microchannel and subsequently to increase their hydrophilicity. Although simple, the effect is unfortunately temporary (i.e. functionalized hydrophilic PDMS will revert back to be hydrophobic in ~8 hours) and not suitable for long-term experimentation. Alternative methods are to silanize the device’s inner walls *in-situ*. However, silanization in microfluidics involving only physical deposition of silane materials will be rapidly degraded by flow shear. In practice, many silanized microfluidics will lose the functionalized layer in ~30 min in shear flows.

In this paper, we employ a technique to coat a durable hydrophilic polyelectrolyte multilayer (PEM) on all inner walls of the device including the PDMS microchannel and glass substrate via an *in-situ* layer-by-layer deposition method^39^ (see methods section for details). This PEM coating permanently reduces the water-air-PDMS contact angle from ~110° to ~20° (Fig. 1b). Due to the aqueous phase having a much higher affinity towards the hydrophilic coating, the walls effectively become oleophobic (Fig. 1d), which is in contrast to an untreated PDMS microchannel (Fig. 1c). Most importantly, we will demonstrate that the functionalized microfluidics can sustain severe flow shear and long-term contact with oil phase for weeks without losing its functionality.

**Figure 2:**
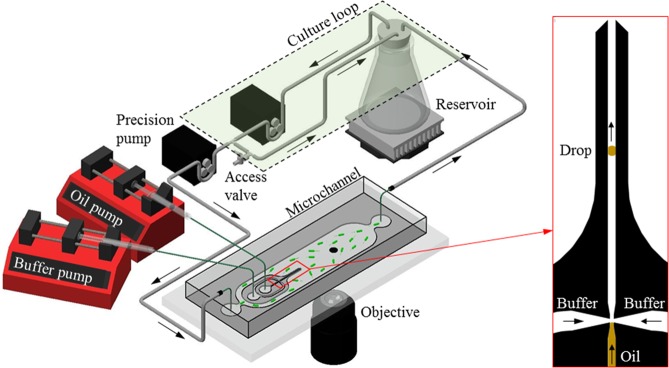
Experimental setup. Particle suspension or microbial culture is contained in the “reservoir”. A peristaltic pump draws the suspension or culture into the recirculating “culture loop” (green box enclosed by dashed lines). The “precision pump” draws sample from the “culture loop” and into the “microchannel” where it encounters the oil drop (dark circle) and observations are made through an “objective” before returning to the “reservoir”. Inset (right): flow focusing junction where a single oil drop is generated.

To form the droplet, we used a simple flow focusing junction wherein oil was essentially pinched from two sides by an aqueous buffer flow to produce a droplet (Fig. 2 inset). Control protocols to facilitate the single droplet generation (Fig. 2) have been developed and discussed in details later. After generation, the droplet was advected through a 200 *μm* wide and 120 *μm* deep nozzle (Inset in Fig. 2) and ejected by the flow into the main channel. The droplet was immobilized by being pinned at the top and bottom of the main microchannel where the microbial interaction experimentation took place (Fig. 2).

#### Immobilization of drop and its hydrodynamics

The most crucial criterion for long term microcosm experimentation is that the droplet must be immobilized in a desired region of the channel and the flow around it must closely emulate that around a rising micro-droplet. Conventional trapping methods (e.g. geometry control^34^, optical tweezers^35^, electrowetting^36^, and acoustic trapping^37^) imposes additional forcing and constraints that cause substantial deviations of flow around a rising oil droplet. Differing from those methods, we immobilized the droplet in a microchannel by directly pinning it at the top and bottom walls. Coincidentally, pinning of the oil droplet can be achieved by the PEM. Initially after the droplet leaves the nozzle (Fig. 2 inset), it moved freely through the 11 mm wide channel while in contact with the top (PDMS) and bottom (glass) walls coated with the PEM. This hydrophilic multilayer is very durable and remains effective after the generation of many droplets under severe flow shear ($> 1000\,{s}^{-1}{\rm{or}} > 4mm\cdot {s}^{-1}$) over days. However, we have found that once the crude-oil droplet remains stationary for an extended period (e.g. from several hours to a day, depending on the specific oil and aqueous phases), it became pinned at polyelectrolyte multilayered surfaces. Once pinned, the contact lines were immobilized and the contact areas between the droplet and the surfaces remained constant. It is particularly worthwhile to emphasize that although pinned, the oil-water-surface contact angles at both walls remained oleophobic, which is evidence that the “pinned” droplet had a maximum circular cross-section at the channel’s mid-plane parallel to the top and bottom walls. The pinning force is sufficiently large to withstand the droplet drag generated by flows at least 10 times as fast as the Stokes rising velocity of a droplet with an equivalent size (e.g. $4mm\cdot {s}^{-1}$ for a 200 *μm* drop). Anecdotally, the oleophobic pinned droplet has been observed in a continuous flow for over one month (results not shown). Note that owing to their small sizes, inertia of micro-droplets is negligible and gravity plays insignificant roles in determining the hydrodynamics around each individual drop. Consequently, flow around a rising micro-droplet can be simulated by an advection flow driven by pressure difference around a stationary droplet in a microchannel.

### Setup and procedure

The close-loop *eChip* platform (illustrated in Fig. 2) including a microfluidic channel, a reservoir, two pumps and connecting tubing to form two recirculating loops (i.e. a primary loop for continuous *in-situ* observations with accurate hydrodynamic conditions and a bypass loop for maintaining suitable chemical and biological microcosm environments) was suitable for a wide range of biotic and abiotic experimentations. To setup a biotic experiment for oil-microbe interaction studies, the entire *eChip* platform was first filled with the sterilized culture medium, after which a single oil droplet was dispensed on-chip and subsequently pinned in the observation area located in the middle of the 11 mm wide microchannel (Fig. 2). The medium was then inoculated with the saturated grown culture through the “access valve”. The culture was allowed to grow *in-situ* to the mid-log growth (or desired growth stage) via the bypass loop, during which time the primary loop remained closed to the microbial suspension. Once the culture reached desired growth condition, the primary loop was activated by a “precision pump” in Fig. 2, enabling real-time observations with controlled flow rates. For an abiotic experiment, the *eChip* was flooded with sterilized solutions (e.g. containing extracellular polymeric substances, EPS, for studying the mechanisms of marine snow formation). After a single stationary droplet was established in the microchannel, the suspension was allowed to interact directly with it. Additional particulates (e.g. latex particles) can also be introduced through the same “access valve” in the return segment of the bypass loop.

**Figure 3:**
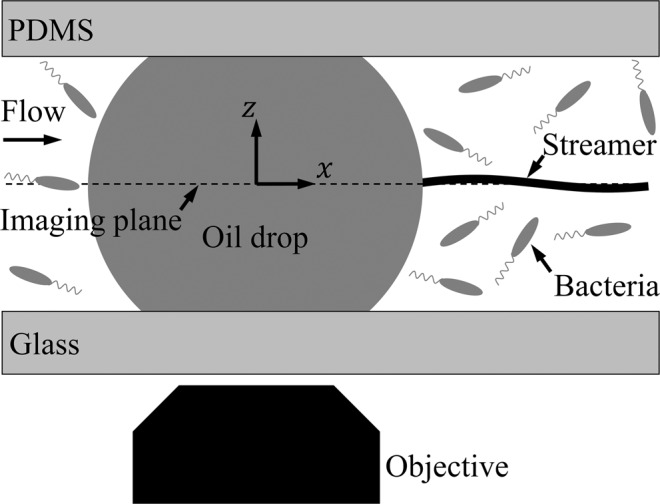
Schematics of ecology-on-a-chip (*eChip*) platform during a microcosm experiment. Side view of a pinned droplet in the microchannel viewed directly by a Nikon microscope.

The real-time interactions were observed by an inverted microscope (Nikon Ti-E, Fig. 3) at 20X magnification (S Plan ELWD Fluor, Depth of Field of ∼5 *μ*m) equipped with a 1K × 1K EMCCD camera (Andor iXon) for time lapse imaging and a 1K × 1K CMOS high speed camera (IDT-NR4) for flow measurement over an area of 720 × 720 *μ*m at resolutions of 0.7 *μm*. With the “time-lapse” camera, images were taken at regular intervals (e.g. 30 s) for the duration of the experiment (e.g. several days or weeks). With the “high-speed” camera, images were recorded at high speed (e.g. 1000 frame s^−1^ for 1 s) at a larger interval (e.g. every 10 min). The time-lapse camera records long term observations of droplet and its morphological changes as well as various phenomena occurring at the oil-water interface, such as microbe adsorption, aggregation and migration, while the high-speed camera allowed for measurements of the flow around the droplet using suspended particles and subsequently evaluated associated hydrodynamics (e.g. drag, viscous stress distributions including normal, shear stresses and pressure).

### Microcosm experiments of microbial interactions with a rising oil droplet

To validate the *ecology-on-a-chip* technique and demonstrate that the technique can obtain previously unattainable observations, we performed kernel experiments on three bacterial isolates (*Pseudomonas sp*. ATCC 27259, *Alcanivorax borkumensis*, ATCC 700651, *Marinobacter hydrocarbonoclasticus*, ATCC 27132) and crude oil (Macondo surrogate). Each isolate was first incubated in its growth medium on a rotary shaker until a stationary growth phase was reached (see Methods). Meanwhile the setup materials were sterilized and assembled, and a droplet was generated and pinned in the observation area of the microchannel (Fig. 2). The sterilized medium (e.g. 50 *ml*) was allowed to circulate through the setup (i.e. both loops) for at least 24 h to verify sterile conditions prior to the inoculation at the beginning of each experiment.

With the precision pump off (Fig. 2, i.e. the primary loop is blocked), the setup was inoculated with the saturated growth culture (e.g. ~1 ml). The culture was then cultivated on-chip through the “culture loop” until it reaches lower mid-log growth (i.e. optical density at 600 nm, $O{D}_{600}$, reaches 0.4). Once the experimental conditions were established, the precision pump was turned on and the bacterial culture begins to flow past the droplet at 2 mm *s*^−1^ (approximately the Stokes rising velocity of a 150 *μ*m oil droplet, *ρ*_oil_ ≈ 850 kg m^−3^, rising in water). Time lapse microscopy at 30 s intervals recorded bacterial aggregation forming directly on the droplet surface.

**Figure 4:**
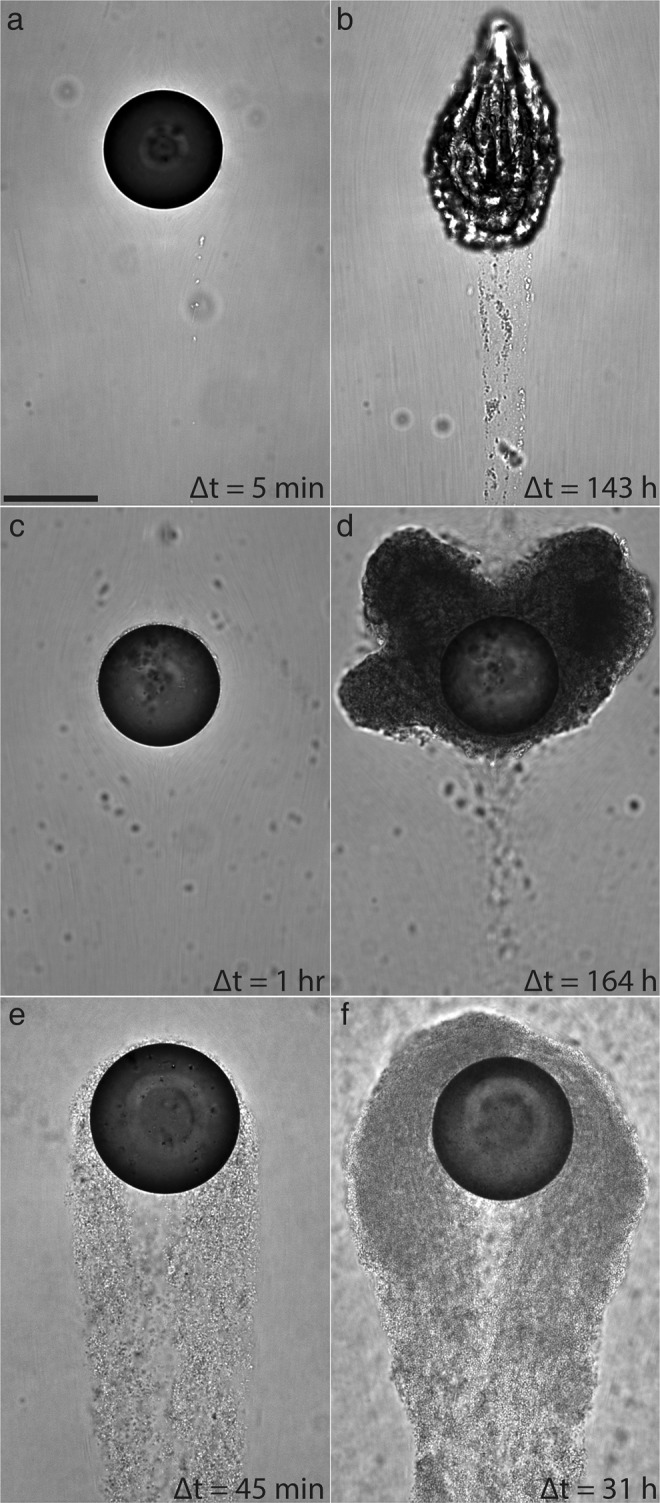
Micrographs of polymeric aggregates formed on an oil droplet by three different bacterial isolates with distinctive differences in aggregate morphology: (**a**,**b**) Alcanivorax borkumensis. (**c**,**d**) Marinobacter hydrocarbonoclasticus, and (**e**,**f**) Pseudomonas sp. (P62), The elapsed time since the first encounter with the bacterial suspension is annotated on the bottom right of each image. Scale: 100 *μ*m.

The time-lapse recordings clearly revealed the formation of polymeric aggregates around a droplet under flow but also the drastic differences in temporal processes and structural characteristics in these aggregates among the three isolates (Fig. 4). Full movies of each time-lapse are available as Supplementary Materials. In the *Alcanivorax* experiment (Fig. 4a,b), filamentous structures consisting of extracellular polymeric substances (EPS) and attached bacteria (i.e. streamers) formed within 30 min, but they were incapable of remaining attached. Meanwhile a thin film of bacteria cell and EPS, with a thickness of up to several cell width (e.g. ~4 *μm*) formed around the entire droplet. Throughout the course of the 95 h movie (Supplemental Material Movie S1) streamers were seen forming, detaching, and forming anew. At the end of the first 24 hours, the droplet appeared to begin wrinkling and then stretches like an elastic sack form the next 3 d with severe deformation^40^.

In contrast, the *Marinobacter* experiment (Fig. 4c,d) developed a film up to ~50 *μ*m thick with a majority of the accumulation at ±45° left and right from the leading stagnation point. In the time-lapse recording (Supplementary Material Movie S2) the film appeared quite malleable and continually flows towards the rear of the droplet. Once collecting at the rear, chunks of the film began sloughing off. The droplet never developed a wrinkly skin like the *Alcanivorax* experiments, and the drop size did not change significantly over the course of 8 d of observation.

In the third experiment, *Pseudomonas* (Fig. 4e,f) formed very rapid streamers within the first hour which were stable enough to grow into a large tail that extends >10 drop diameters downstream. After 14 h, the tail began to disperse both through shear erosion along the outside and central hollowing in the inside (Supplementary Material Movie S3). At the end of the first day the film grew back denser and stiffer and a long tail reformed, indicating some kind of “life cycle” of the *Pseudomonas* aggregate. These three examples demonstrate wildly different aggregation behavior and only scratches the surface of the extremely complex microbiological processes at a rising oil droplet.

### Flow measurements around a “rising” droplet

**Figure 5:**
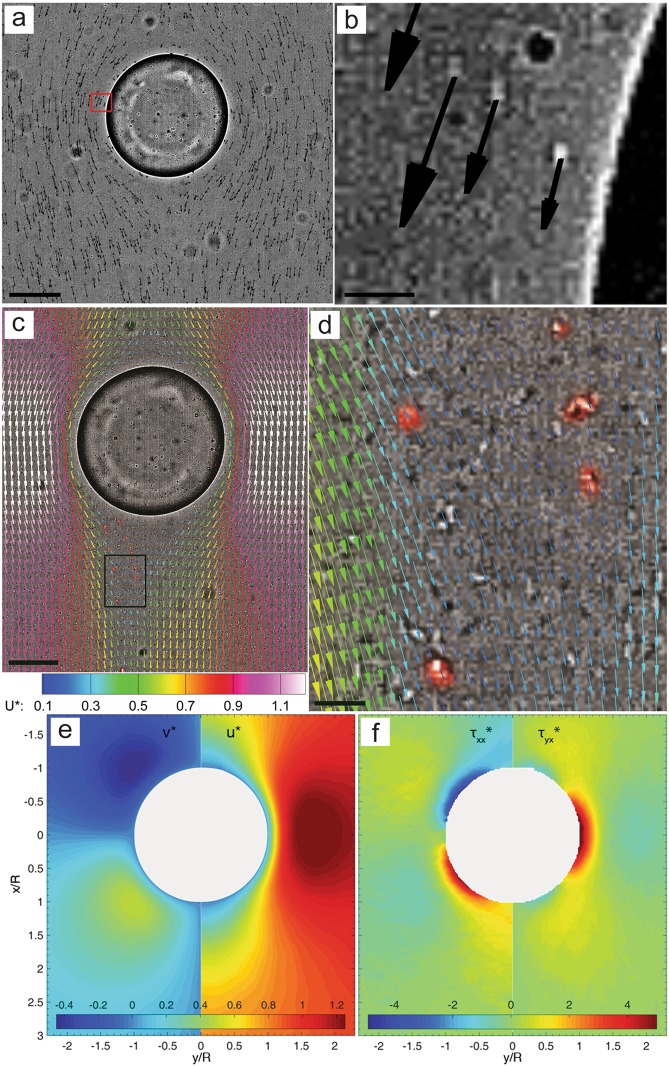
Characterization of hydrodynamic flow around a rising oil droplet with extracellular polymeric substance aggregation with eChip platform. (**a**) a single frame from a high speed image sequence showing bacterial motion. Arrow: cell displacement. (**b**) Close-up of (**a**) showing cell velocity near the drop surface. Bright rod-like spot: an in-focus image of Pseudomonas cell. (**c**) Flow velocity map around a rising droplet with two trailing “streamers”. The velocity vectors are overlaid with a single image frame. Only every 5^th^ and 7^th^ vector is shown in the cross-flow (y) and streamwise (x) direction, respectively. Color: Velocity magnitude. (**d**) Close-up of flow map at the region near two “streamers”, marked by black rectangle in (**c**). Here every vector in y axis and every 2^nd^ vector in x-axis is shown. Bacteria “trapped” along two separate transparent EPS streamers are highlighted in a pseudo color, red. (**e**) Normalized velocity magnitude map. Left-half: cross-stream (*v*), Right-half: streamwise (u^*^) components. (**f**) Normalized viscous stresses. Left-half: streamwise normal stress, ${{\rm{\tau }}}_{{\rm{xx}}}^{\ast }={\partial }^{\ast 2}{{\rm{u}}}^{\ast }/{({\partial }^{\ast }{{\rm{x}}}^{\ast })}^{2}$, Right-half: shear stress, ${{\rm{\tau }}}_{{\rm{xy}}}^{\ast }=[{\partial }^{\ast 2}{{\rm{u}}}^{\ast }/{({\partial }^{\ast }{{\rm{y}}}^{\ast })}^{2}+{\partial }^{\ast 2}{{\rm{v}}}^{\ast }/{({\partial }^{\ast }{{\rm{x}}}^{\ast })}^{2}]/2$. Scale: (**a**,**c**,**d**) 100 μm, (**b**,**d**) 10 μm.

In addition to aggregate morphology, this *ecology-on-a-chip* system is capable of providing highly resolved simultaneous flow measurements for providing quantitative insights into hydrodynamic impact of colloidal aggregates on the transport of oil droplets (e.g. rising velocity). To demonstrate, we performed experiments using the *Pseudomonas sp*. from the previous section with the same experimental procedure. In addition to the time-lapse recording (shown above), we recorded images at 1000 fps for a 1 s period at intervals of 10 min. The individual suspended bacteria were used as flow tracers and their positions were tracked from frame to frame to produce the displacement of microbes (Fig. 5a,b). Using particle tracking velocimetry (PTV) (see Methods for details), the instantaneous velocity maps allowing flow measurement very close to the interface (Fig. 5a) were obtained. This near interface measurement capability is highlighted in Fig. 5b, i.e. the closest velocity measurement was located in ~2 cell body length closest to the interface. Each high speed sequence of 1000 images produced ∼3 × 10^6^ unstructured velocity vectors per time step, which were interpolated onto structured grids to produce highly resolved (e.g. a vector spacing of 2 um) velocity map (Fig. 5c,d). Note that vectors in Fig. 5c are shown one in every seven in the streamwise (*x*) direction and one in every five in the transverse (*y*) direction. The unprecedented measurement resolution is highlighted in Fig. 5d showing flow near nanometer polymeric filaments (marked by sporadically attached bacterial cells with red). The high resolution measurements allowed direct assessment of budgets in streamwise momentum balance: $R{e}_{{D}_{d}}({\overrightarrow{u}}^{\ast }\cdot {\overrightarrow{\nabla }}^{\ast }){\overrightarrow{u}}^{\ast }+{\overrightarrow{\nabla }}^{\ast }{p}^{\ast }-{\overrightarrow{\nabla }}^{\ast }\cdot {\overrightarrow{\nabla }}^{\ast }{\overrightarrow{u}}^{\ast }=0,$ where the superscript “*” denotes the normalized quantities or operators and “$\nabla$” is the gradient operator, as well as $R{e}_{{D}_{d}}$ is a dimensionless parameter based on droplet diameter (${D}_{d}$). Lengths are scaled by ${D}_{d}$, velocities by incoming flow velocity (${U}_{f}$), and stresses by ${\mu }_{f}{U}_{f}/{D}_{d}$ where ${\mu }_{f}$ is fluid viscosity. Normalized velocity magnitudes are shown in Fig. 5e, and viscous stresses (${\overrightarrow{\nabla }}^{\ast }\cdot {\overrightarrow{\nabla }}^{\ast }{\overrightarrow{u}}^{\ast }$) in Fig. 5f, from which the elusive pressure gradients can be approximated.

## Conclusion

We present an experimental framework that to-date provides the closest emulation of the chemical, biological, and physical environments associated with active colloids, e.g. microbes, and oil-water interfaces. Specifically, we have demonstrated the above methods can simulate an oil droplet, e.g. 100 *μ*m, rising through a microbial suspension over a timescale of weeks, while providing direct uninterrupted microscopic observation of bacteria at the oil-water interface, a previously unattainable task. The experimental design overcome several hurdles:

1. A single oil droplet is generated on-chip in an aqueous continuous phase, a non-trivial feat due to the oil’s strong affinity to spread over the inherently hydrophobic PDMS channel. This is overcome by first depositing a robust hydrophilic polyelectrolyte multilayer (PEM) to the channel walls using layer-by-layer deposition;
2. The oil droplet, although initially freely flowing through the channel, has its contact line pinned while preserving the oleophobic contact angle with both walls. While further investigations of this are planned, it is suspected that the crude oil over time degrades/penetrates the PEM, locally adhering to the channel walls only within the contact area;
3. The oleophobic contact angle between the oil and the channel walls, and thus the curved oil droplet interface, can be preserved for over a month due to the stability of the PEM and resistance to degradation by chemical, biological, and physical process during the experiment. This is in contrast with short-lived plasma-functionalized channels and easily-sheared silanized channels.

Together with an automated microscope platform and synchronized time-lapse and high-speed image capturing, first of their kind long-term observations of colloidal interaction and aggregation at an oil-water interface under flow shear with periodic high-speed imaging to provide flow field measurements are possible. This was demonstrated by three kernel experiments consisting of bacterial isolates *Pseudomonas sp*., *Alcanivorax borkumensis*, and *Marinobacter hydrocarbonoclasticus*, all relevant to marine environments. The drastic differences in aggregate morphology and interfacial response between the three isolates is striking in Fig. 4, and suggests a rich future for this experimental platform to delve into the essentially unexplored wide array of microbial responses to oil-water interfaces. This is considerably enhanced by the additional flow field measurements (Fig. 5) which allow for elusive quantification of the physical impact of these active colloids at sheared oil-water interfaces.

## Materials and Methods

### Fluidics circuit

The fluidic circuit consists of a 125 ml flask reservoir, a 12 V 50 rpm peristaltic pump (INTLLAB), a micro-peristaltic pump (marked as “precision pump” in Fig. 2, Masterflex C/L, Cole-Parmer), and a microfluidic channel (details below). These components are interconnected using soft ¼” OD, 3/16” ID Tygon tubing (Cole-Parmer) with 1/16” OD PEEK tubing (IDEX) used to connect to microfluidic channel. Proper PEEK fittings (IDEX) connect the PEEK tubing to polypropylene barbed fittings (Cole-Parmer) connected to the Tygon tubing. Two flow loops are constructed as shown in Fig. 2; one “culturing loop” which bypasses the microchannel, and the observation loop which does flow through the channel. A polycarbonate stopcock is connected to the “culturing loop” to provide access to the fluidic circuit for either inoculation or sampling. A chemostat can also be integrated into the circuit to control microbe concentrations in the *eChip* platform in conjunction with the reservoir.

### Microfluidic channel and fabrication

The channel is symmetric with the height of 100 *μ*m, the length of 60 mm, and the width of 11 mm. There are four fluid ports with the diameter of 1.5 mm: two primary ports for circulating the bacterial suspension through the microchannel during microcosm experiments, and two auxiliary ports, *i*.*e*. one for the oil inlet, and one for the aqueous buffer inlet used for generating a single oil droplet. PEEK tubing ($OD=1/16\mbox{''}$) interfaces directly with the ports. A co-axial nozzle with flow-focusing junction (Inset in Fig. 2) generates a single oil droplet. The nozzle has a width of 100 *μ*m and the narrowest cross-section of the junction is 50 *μ*m. The dispensing procedure is detailed below.

The channel is made of poly(dimethylsiloxane) (PDMS, Dow Corning) and fabricated using conventional soft lithography^41^. A hard chrome mask of the 2D channel layout is made using a mask writer (Heidelburg). On a 4 in silicon wafer, a 100 *μ*m thick layer of SU-8 negative photoresist (SU-8 2075, MicroChem) is spin-coated at 2200 rpm for 30 s, soft baked on a hotplate at 65 °C for 5 min, and then hard baked at 95 °C for 20 min. The wafer is then patterned using a mask aligner (Carl Suss) for 30 s with hard contact mode. A post-exposure bake at 65 °C for 5 min and followed by 95 °C for 10 min. The master is developed in 1-methoxy-2-propanol acetate (Fisher) for 17 min at room temperature to reveal the channel features. The baking protocols must be strictly followed to prevent thermal induced cracks often developed at feature with sharp corner such as flow focusing junction and nozzle tips.

PDMS is mixed at a ratio of 10:1 (PDMS: cross-linking agent) and degassed in a desiccator. The mixture is poured onto the master and cured in an oven at 65 °C for 2 days. After curing, the PDMS channel is cut and released from the master, as well as fluid ports are produced using a 1.5 mm biopsy punch. A 1″ × 3″ microscope slide is cleaned with “piranha” etching solution (98% H_2_SO_4_ and 30% H_2_O_2_ at a ratio of 2:1 v/v) and bonded with the PDMS channel by exposing to air plasma for 1.5 min in a plasma cleaner (Harrick). Immediately after bonding the hydrophilic layer-by-layer functionalization procedure follows.

### Layer-by-layer hydrophilic polyelectrolyte coating

**Figure 6:**
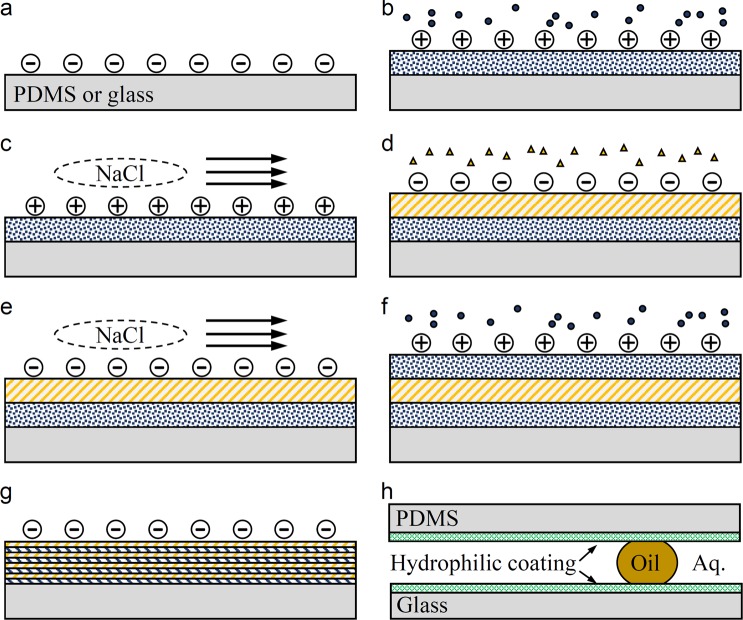
Schematic of the layer-by-layer hydrophilic functionalization process. (**a**) PDMS and glass surfaces (solid gray) are activated using air plasma. (**b**) PAH is injected into the channel and adsorbed to walls, while unabsorbed PAH remains suspended (circles). (**c**) The channel is rinsed with NaCl solution to remove the suspended PAH. (**d**) PSS is injected and adsorbed to the PAH layer, while unabsorbed PSS remains suspended (triangles). (**e**) NaCl solution wash removes unadsorbed PSS. (**f**) A second layer of PAH is adsorbed to the first PSS layer. (**g**) A completed multilayer is deposited with PSS as the final layer. (**h**) The hydrophilic walls effectively create an oleophobic microfluidics.

The PDMS-glass microchannel walls are functionalized to be hydrophilic with a layer-by-layer deposition technique^39^. Figure 6 provides a schematic representation of the functionalization process. Immediately after bonding the PDMS microchannel to the glass substrate with air plasma, the device is filled with a 0.5 M NaCl (Sigma-Aldrich) solution containing 10 *μ*M poly(allylamine hydrochloride) (PAH, Alfa Aesar) with a molar mass of 120,000–200,000 g mol^−1^. The PAH adsorbs to the negatively charged channel walls, producing a positively charged PAH layer. After 5 min, the PAH is removed and the channel is rinsed thoroughly with 0.1 M NaCl to remove any remaining unadsorbed PAH. The device is then filled with a 0.5 M NaCl solution containing 10 *μ*M poly(sodium 4-styrenesulfonate) (PSS, Sigma-Aldrich) with a molar mass of 1,000,000 g mol^−1^. The PSS adsorbs to the positively charged PAH layer to form a negatively charged PSS layer. After 5 min, the PSS is removed and the channel is rinsed thoroughly with 0.1 M NaCl solution to remove any unadsorbed PSS. The washing step is crucial due to the tendency of PSS and PAH to form precipitates. This process is continued with alternating PAH/PSS deposition with NaCl washes in between until multiple PAH-PSS layers (e.g. 4 layers are used in experiments) are formed with PSS as the final layer. The channel is then rinsed thoroughly with DI water (Millipore Milli-DI) and dried in a desiccator. Anecdotally, the functionalization remains effective after exposure to severe flow shear for weeks. The shelf-life of the functionalized surface lasts at least 5 months when stored in vacuum.

### Dispensing and pinning single droplet

The droplet is dispensed by manually controlling syringe pumps (New Era Pump Systems) for the oil and the aqueous buffer (Fig. 2). A glass syringe (Hamilton Gastight) is used for oil, while a 3 ml disposable syringe (BD) is used for buffer solution. With the “precision pump” (a Masterflex C/L peristaltic pump, Cole-Parmer) operating at a flow rate of 148 *μ*l min^−1^, the buffer solution is injected into microchannel at 50 *μ*l min^−1^ while the oil is infused at 100 nl min^−1^. At this low flow rate, the oil slowly approaches the flow-focusing junction (Fig. 2 inset). As the oil enters the junction, the syringe pump for oil is turned off. The oil plug will neck with the aid of two side impinging flows and subsequently pinches off to form a single droplet. Once pinched off, the droplet is advected downstream along the nozzle. After exiting the nozzle into the 11 mm wide observation channel, the droplet is further carried downstream by the flow generated by the Masterflex pump. Once the droplet reaches the center of the observation area in the main channel, both syringe pump for buffer solution and Masterflex peristaltic pump for culturing fluids are turned off. The droplet will subsequently come to rest. Left in this state overnight, the droplet becomes pinned (or immobilized) to the top (PDMS) and bottom (glass) channel walls while preserving its oleophobic contact angle with the walls as well as its circular cross-section.

### Sterilization

All materials are autoclaved at 121 °C for 30 min except for the polycarbonate stopcock (“access valve” in Fig. 1) and the microchannel. The stopcock is soaked in 70% ethyl alcohol for 30 min and left to dry in a laminar flow hood with UV light. The functionalized microchannel is filled with 70% ethyl alcohol for 30 min, drained, and left to dry in the laminar flow hood. Culture media used to fill the fluidics circuit prior to inoculation is autoclaved. The experimental components are assembled in the laminar hood and then carefully transferred to the microscope. Following dispensing and pinning of the droplet, the sterile culture medium is circulated through the entire fluidic circuit including both loops for at least 24 hr to verify sterile conditions. Note that this sterilization procedures are rigorously followed before every experiment.

### Culturing

In this manuscript, bacteria used are *Alcanivorax borkumensis* (ATCC 700651), *Marinobacter hydrocarbonoclasticus* (ATCC 27132), and *Pseudomonas sp*. (ATCC 27259). The microbes are cultured according to a two-step growth protocol. First the bacteria are grown on a rotary shaker at 120 rpm and room temperature in ATCC-recommended growth media: Difco Marine Broth 2216 (BD) (37.4 g L^−1^) with sodium pyruvate (Fisher) (10 g L^−1^) for *Alcanivorax*. Difco Marine Broth 2216 (37.4 g L^−1^) without sodium pyruvate for *Marinobacter*, Difco Nutrient Broth (BD) (8 g L^−1^) for *Pseudomonas*. In each culture, 20 ml of the respective medium is inoculated with 100 *μ*l of corresponding short term stock stored at −20 °C. The cultures are allowed to reach saturation on a shaker at 20 °C (typically it takes ~4 d for the first growth).

While the first growth is on-going, the experimental setup is filled with 50 ml of sterile culture medium and an oil droplet is dispensed and pinned as described above. After verifying sterile conditions, and with the “precision pump” (Fig. 2) off, the medium is inoculated with 100 *μ*l of the saturate culture grown on the shaker through the sterilized “access valve” (Fig. 1). The inoculated culture circulates in the “culture loop” (Fig. 2) isolated from the microchannel until the optical density at 600 nm wavelength is ${{\rm{OD}}}_{600}=0.4$. Once the desired ${{\rm{OD}}}_{600}$ is reached, the “precision pump” is turned on to flow the microbial culture into the microchannel and the experiment begins.

### Image acquisition

Observations are made using a Nikon Ti-E microscope with Nikon 20X S Plan Fluor ELWD objective and differential interference contrast (DIC) microscopy. Two cameras operate simultaneously to record time-lapse images as well as high speed images. Through the left camera port, a 1K × 1K EMCCD camera (Andor DU-888) records images every 30 s for the duration of the experiments which are streamed directly to a data storage (24TB data server). Through the right camera port, an 1K × 1K CMOS high-speed camera (IDT NR4S) records images at 1000 fps for 1 second period every 10 min such that at least 1000 images are recorded per period. A custom MATLAB script prompts the microscope to automatically switch between ports and synchronize cameras allowing experiments to run unattended.

### Image analysis and flow measurements

High speed images acquired with the IDT NR4S CMOS camera are used to obtain flow measurements using micro-particle image velocimetry (*μ*PIV)-assisted particle tracking velocimetry (PTV). The measurement area is 720 × 720 *μ*m using the Nikon Ti-E microscope with a 20X objective, and the depth of field is 5 *μ*m so flow measurements are averaged over a 5 *μ*m thickness. The microscope is focused at the mid-plane of the channel. The suspended bacteria cells (or other particles if applicable) are used as flow tracers. Since *μ*PIV techniques have been used extensively in the literature, we provide a concise summary here. Following image acquisition, every two consecutive images in the sequence undergo conventional cross-correlation PIV analysis^42^. For a given frame, the bacterial cell locations are determined and in the following frame their new positions are found with the assistance of the PIV velocity map i.e. PIV-assisted PTV^43^. Thus for each cell location in an image, a velocity vector is found. With bacterial concentrations approaching ~1 × 10^6^ cell ml^−1^ typically on the order of ~1000 cells are located in a frame. Over the entire sequence an order of ~1 × 10^6^ velocity vectors are measured which are mapped onto a 4 pixel (2.7 *μ*m) grid using a Taylor expansion scheme^43^.

## Supplementary information

Supplemental Information

Video S1

Video S2

Video S3
