# Supplementary material for: A new ecology-on-a-chip microfluidic platform to study interactions of microbes with a rising oil droplet
Source: Sci Rep. 2019 Sep 24;9:13737. doi: 10.1038/s41598-019-50153-9 (PMC6760120; doi:10.1038/s41598-019-50153-9)
Supplement: Supplementary file 1 — Supplemental Information [file 41598_2019_50153_MOESM1_ESM.pdf]

Supplemental Information:

A new *ecology-on-a-chip* microfluidic platform to study interactions of microbes with a rising oil droplet

by Andrew R. White[1], Maryam Jalali[1], Jian Sheng[1*]

[1]Department of Engineering, Texas A&M University–Corpus Christi, Corpus Christi, TX 78412, USA

Movie Legends

Video S1. A time lapse video of the experiment using *Alcanivorax borkumensis* corresponding to Fig. 4a. The scale bar is 100 μm and the time since initial exposure to the bacteria is shown in the top left.

Video S2. A time lapse video of the experiment using *Marinobacter hydrocarbonoclasticus* corresponding to Fig. 4b. The scale bar is 100 μm and the time since initial exposure to the bacteria is shown in the top left.

Video S3. A time lapse video of the kernel experiment using *Pseudomonas* corresponding to Fig. 4c. The scale bar is 100 μm and the time since initial exposure to the bacteria is shown in the top left.
